# An Evidence-Based Framework for Creating Inclusive and Personalized mHealth Solutions—Designing a Solution for Medicaid-Eligible Pregnant Individuals With Uncontrolled Type 2 Diabetes

**DOI:** 10.2196/46654

**Published:** 2023-10-12

**Authors:** Naleef Fareed, Christine Swoboda, Yiting Wang, Robert Strouse, Jenelle Hoseus, Carrie Baker, Joshua J Joseph, Kartik Venkatesh

**Affiliations:** 1 Department of Biomedical Informatics College of Medicine The Ohio State University Columbus, OH United States; 2 Center for the Advancement of Team Science, Analytics, and Systems Thinking College of Medicine The Ohio State University Columbus, OH United States; 3 Department of Research Information Technology College of Medicine The Ohio State University Columbus, OH United States; 4 Health Impact Ohio Columbus, OH United States; 5 Division of Endocrinology, Diabetes and Metabolism College of Medicine The Ohio State University Columbus, OH United States; 6 Division of Maternal-Fetal Medicine Department of Obstetrics and Gynecology The Ohio State University Columbus, OH United States

**Keywords:** personalization, mobile health, mHealth, pregnancy, pregnant, maternal, personalized, diabetic, algorithm, diabetes, rule-based algorithms, social determinants of health, inclusive, inclusivity, design

## Abstract

Mobile health (mHealth) apps can be an evidence-based approach to improve health behavior and outcomes. Prior literature has highlighted the need for more research on mHealth personalization, including in diabetes and pregnancy. Critical gaps exist on the impact of personalization of mHealth apps on patient engagement, and in turn, health behaviors and outcomes. Evidence regarding how personalization, engagement, and health outcomes could be aligned when designing mHealth for underserved populations is much needed, given the historical oversights with mHealth design in these populations. This viewpoint is motivated by our experience from designing a personalized mHealth solution focused on Medicaid-enrolled pregnant individuals with uncontrolled type 2 diabetes, many of whom also experience a high burden of social needs. We describe fundamental components of designing mHealth solutions that are both inclusive and personalized, forming the basis of an evidence-based framework for future mHealth design in other disease states with similar contexts.

## Introduction

Mobile health (mHealth) apps have the potential to be an evidence-based approach to improve health behavior and outcomes. Some research studies have demonstrated that mHealth can improve health care access and delivery, adherence to care regimens, and patient self-management and behavior modifications for various chronic conditions, including type 2 diabetes (T2D), asthma, obesity, and hypertension [1-3]. A recent systematic review of mHealth use for diabetes (both type 1 diabetes and T2D) and obesity concluded that mHealth had a beneficial effect on outcomes, including hemoglobin A_1c_ (HbA_1c_) reduction (–0.3% to –0.5%). The review, however, noted that personalizing different components of mHealth to end users was particularly an unaddressed need [4].

An app, for example, can personalize educational content to a patient based on their clinical severity, lifestyle preferences, and social needs (eg, safe housing). Personalization can be abstractly defined as a “process which changes the functionality, interface, content, or distinctiveness of a system to increase its personal relevance” [5]. Prior research and theoretical frameworks have emphasized that personalization is an important determinant of engagement, which in turn is a determinant of health outcomes [6-8]. We define engagement in the context of this paper as patient interaction and behavior with a mHealth app and their care, including actions such as time spent with the app and using information from the app to communicate with care team members.

There are important theoretical and practical knowledge gaps existing in the link among mHealth personalization, engagement, and health outcomes for pregnant individuals living with diabetes*.* Evidence gaps exist in how these 3 areas could be aligned when designing mHealth apps for populations that experience significant adverse social determinants of health (SDoH) burden, including Medicaid-insured pregnant individuals with poor glycemic control [9,10]. In the spirit of Eyles et al [11], our paper collectively integrates various aspects of user-centered design (UCD) in mHealth tools that have been previously studied in silos. We use our ACHIEVE (successfully achieving and maintaining euglycemia during pregnancy for type 2 diabetes through technology and coaching) solution as the basis to address these knowledge gaps by proposing an evidence-based framework for designing mHealth apps that is both inclusive and personalized. This framework demonstrates how the design, implementation, and evaluation of such tools, driven by the principles of UCD, can be collectively undertaken for any digital health tool.

The ACHIEVE solution focuses on the development of a mHealth solution to improve glycemic control for Medicaid-eligible pregnant individuals with uncontrolled T2D. Briefly, our ACHIEVE solution is a multicomponent system, including a mHealth app, provider dashboard, continuous glucose monitoring (CGM), and care team coaching for medical and social needs [12]. This solution empowers Medicaid-eligible pregnant individuals with T2D to achieve glycemic control, improve access to care, and acquire patient education and support. Each subcomponent of the proposed solution is grounded in Social Cognitive Theory, and emphasizes an individual’s skills, knowledge, beliefs, and self-efficacy to achieve glycemic control [13,14]. Current mHealth apps designed for individuals with T2D outside of pregnancy perform well on education and information functions, but poorly on engagement [4,15]. Few existing apps in both pregnancy and diabetes provide comprehensive evidence-based educational content, tracking tools, UCD, and integration with electronic health records or CGM data to support care team monitoring [15,16].

The novelty of our approach with the ACHIEVE mHealth solution addresses 2 specific areas: personalization with a clinically high-risk population and closing digital health inequities that exist among specific underserved populations, such as pregnant individuals who experience a high burden of unmet social needs. Our experiences with designing a personalized mHealth solution with a diverse high-risk patient population could be adapted to similar populations and contexts. Based on our approach, we describe individual components of designing mHealth solutions that form the basis of an evidence-based framework for future mHealth apps that are both inclusive and personalized. We first provide background information on the ACHIEVE solution and then describe its components within the context of the study population.

## Background

### Pregnancy With T2D

Poorly controlled T2D increases the risk of adverse pregnancy outcomes for the mother and the infant, including severe maternal morbidity, fetal growth abnormalities, preeclampsia, preterm birth, and neonatal intensive care unit admission [17-20]. Glycemic control decreases the risk of these complications [17-19]. The increasing insulin resistance during pregnancy in addition to experiencing a high burden of social needs (eg, inadequate transportation, food insecurity, and lack of access to quality health) care make it difficult to maintain glycemic control in pregnancy [20]. Medicaid-insured pregnant individuals with T2D are less likely to access preconception care and have worse glycemic control as measured by HbA_1c_, and hence, are more likely to experience adverse pregnancy outcomes [21]. The clinical management of T2D in pregnancy includes strict and maintained pharmacotherapy and lifestyle changes to maintain glycemic control [22]. Medicaid-insured pregnant individuals with T2D experience significant barriers to successful diabetes management, including self-monitoring of blood glucose (SMBG), frequent changes in insulin dosing, inadequate resources to adhere to prescribed treatment plans, and a high burden of unmet social needs [23-25]. Disparities arising from these challenges manifest through inadequate glycemic control, lack of attendance at prenatal visits, insufficient engagement with care plans, and treatment nonadherence [26,27].

### Multimodal Data Integration for Pregnant Individuals Who Have Social Needs and Are Living With T2D With Poor Glycemic Control

Patient-reported outcomes (PROs) that capture both physical and psychological symptoms can support the delivery of more patient-centered care [28]. The collection of PROs allows for ecological momentary assessment, which facilitates repeated assessments of patients [29,30]. Our prior research has indicated that most patients in our study population do not adequately and accurately complete their glucose logs based on SMBG [12]. Integration of PROs into prenatal care delivery has the potential to improve glycemic control for Medicaid-eligible pregnant individuals with T2D [31]. Such an approach can elucidate problems in achieving and maintaining glycemic control, allow for monitoring T2D self-care, improve patient-care team communication, and enable successful T2D self-management. Many of these tasks can be seamlessly performed by a CGM device that can be linked to a mHealth app and provider dashboard. Advances in CGM can better characterize the glycemic response of pregnant individuals with T2D by identifying individualized glycemic patterns [32,33].

T2D in pregnancy disproportionately affects Medicaid-eligible individuals who are more likely to experience a high burden of social needs [34]. Outside of pregnancy, individuals with T2D who experience more social needs are at higher risk of complications resulting from inadequate glycemic control [35,36]. T2D management in pregnancy is expensive, with >US $7000 in excess pregnancy-related costs alone [37]. Over 40% of pregnant individuals in the United States receive prenatal care through public health insurance such as Medicaid [38,39]. Early and maintained prenatal care improves pregnancy outcomes for T2D [40]. Medicaid-eligible pregnant individuals encounter multiple barriers including unmet social needs that preclude timely diabetes and prenatal care [24,41]. Based on the determinants of health model, clinical care alone for this high-risk population is not sufficient, given the extent to which social needs influence health outcomes [42]. Social needs, such as food security, adequate housing, a safe environment, and access to health care, influence health outcomes and glycemic control [19]. When social needs are not met, T2D management becomes increasingly difficult [36,43].

### An Integrated mHealth Solution for Medicaid-Eligible Pregnant Individuals With T2D That Is Inclusive and Personalized

mHealth can be used as part of a multicomponent solution to achieve glycemic control for Medicaid-eligible pregnant individuals with T2D and poor glycemic control. Mobile phone ownership exceeds 90% among reproductive-age Medicaid-enrolled women [44]. mHealth apps can improve timely care delivery, tailor patient education and support, and provide convenient communication between the patient and care team [45]. Pregnant individuals are interested in engaging in alternative prenatal care models [46], which can reduce racial and ethnic disparities in pregnancy outcomes [41]. In addition, a linked provider dashboard can facilitate regular contact between patients and providers and improve patient outcomes [47]. The value of mHealth apps for health-based solutions in diabetes outside of pregnancy has been demonstrated [48,49], and results in improved HbA_1c_, more judicious health care usage, better PROs [50-53], and better postintervention patient engagement [54]. There is a lack of apps that encompass all of the functions necessary to improve pregnancy and diabetes care and management, so people in this population may have to separately manage these 2 complex conditions using multiple apps or systems. In addition, management needs for those with only T2D may differ, as diet, blood glucose, and medication recommendations will not be considering the needs of a growing pregnant woman and fetus.

CGM makes it easier for individuals to monitor their glucose values without needing to check finger stick values and may also detect periods of elevated glucose values, such as postprandial blood glucose levels and subclinical nocturnal hypoglycemic events, that are associated with adverse pregnancy outcomes [55,56]. For pregnant individuals with type 1 diabetes, research has demonstrated that the use of CGM rather than SMBG improves neonatal outcomes [57]. However, CGM has not been widely tested for pregnant individuals with T2D. Our prior work demonstrated that Medicaid-eligible pregnant individuals with T2D prefer CGM to SMBG [12]. When provided with CGM, pregnant individuals with T2D express high levels of satisfaction [58]. Patients are more likely to benefit from mobile technology if they understand CGM data, meaningfully displayed through an app, and how to actively respond to it to achieve their glycemic goals [59-62].

Integrating a mHealth app with a provider dashboard can enhance team-based coaching and patient engagement. Few existing apps provide comprehensive evidence-based educational content, tracking tools, UCD, and integration with electronic health records or CGM [15,16]. The UCD approach specifies the needs of end-users, and involves end-users in a co-design process to develop mHealth apps that meet their requirements and represents a digitally inclusive approach to using mHealth apps [63]. mHealth apps that are comprehensive, personalized, and integrated within care team workflows are more likely to be effective [15], increase patient uptake [64], and maintain patient and provider engagement over time [64,65]. Provider-facing bidirectional dashboards linked to data from the mHealth app can offer comprehensive diabetes information to care teams, including timely clinical alerts about glycemic control, psychosocial issues, and treatment plans [66]. Dashboards can facilitate team-based provider coaching and feedback [67], including working with the patient's agenda, recognizing patient beliefs, values, and readiness for change; and helping with behavioral modification [68,69]. Outside of pregnancy, such an approach has improved glycemic control among adults with T2D [48] and adherence to evidence-based diabetes care among young Latinx youths living with T2D [66].

In sum, the motivation behind the development of our ACHIEVE solution was a call to action for our team to build multifaceted systems around mHealth apps that manifest as “digital ecosystems” within which patients engage with their health and their care. We present the key components of the design of this multifaceted solution below.

## Evidenced-Based Framework for mHealth Personalization

### Overview

**Figure 1:**
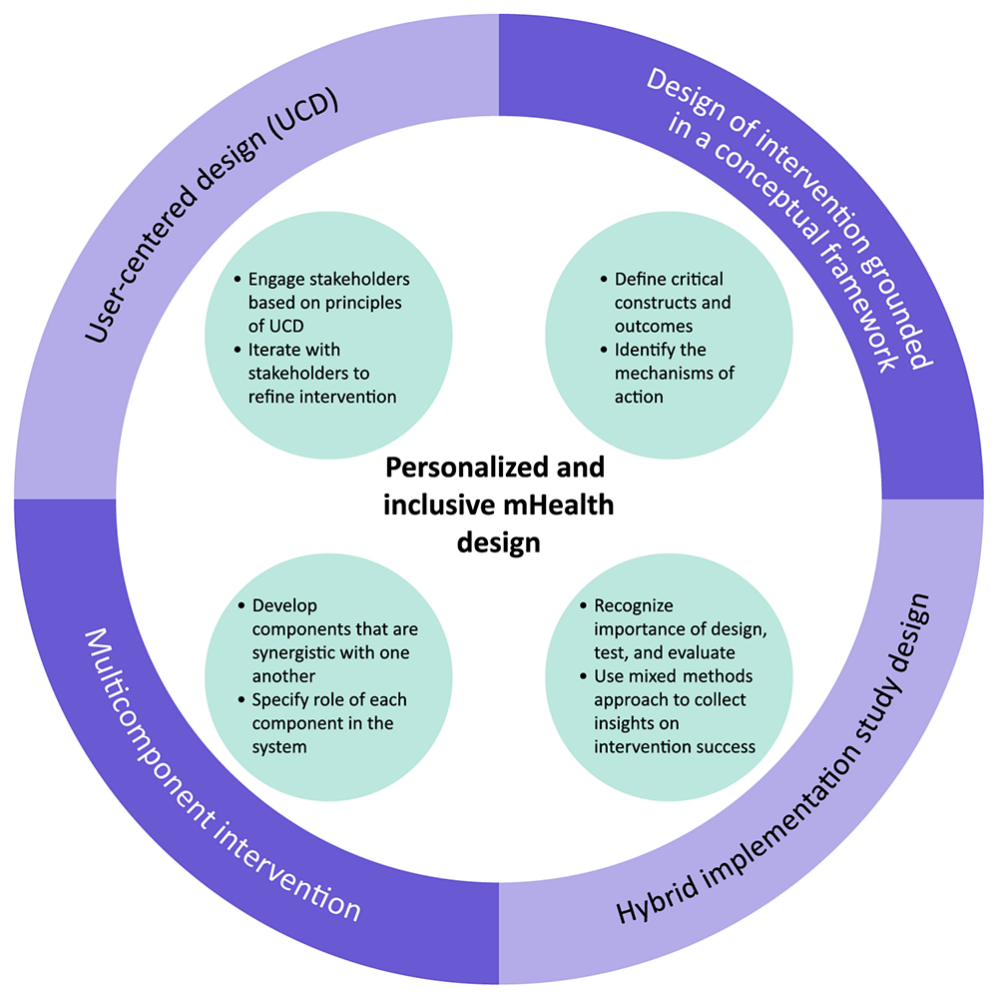
Evidence-based framework to design mHealth apps that are both inclusive and personalized. mHealth: mobile health.

We identified 4 components (Figure 1) that are vital for the design of the ACHIEVE solution and represent areas of consideration for designing mHealth apps that are both inclusive and personalized, especially for populations such as Medicaid-enrolled pregnant individuals with uncontrolled T2D. We describe each component below for our proposed evidence-based framework. Evidence collected for the development of the ACHIEVE solution and our framework is based on research activities approved by our institutional review board.

### Focus on Key Stakeholders by Being Inclusive and Using UCD

In a UCD approach, end users influence the final design. End-user involvement in the design process leads to safer, more efficient, and more effective products with greater acceptance and success [63,70]. Recent research among publicly insured populations highlights the importance of UCD as a means to extend the benefits of digital health technologies such as mHealth to clinically high-risk populations who experience a high burden of social needs [10].

We established a user-centered design work group (UCDWG) composed of 10 members from three stakeholder groups: (1) health care providers (physicians and certified diabetes care and education specialists [CDCES]) who care for pregnant individuals with T2D; (2) Medicaid-eligible individuals with T2D who have previously given birth; and (3) community-based stakeholders, including community health workers (CHWs) and licensed social workers. The purpose of the UCDWG was to guide the adaptation and refinement of the solution.

**Figure 2:**
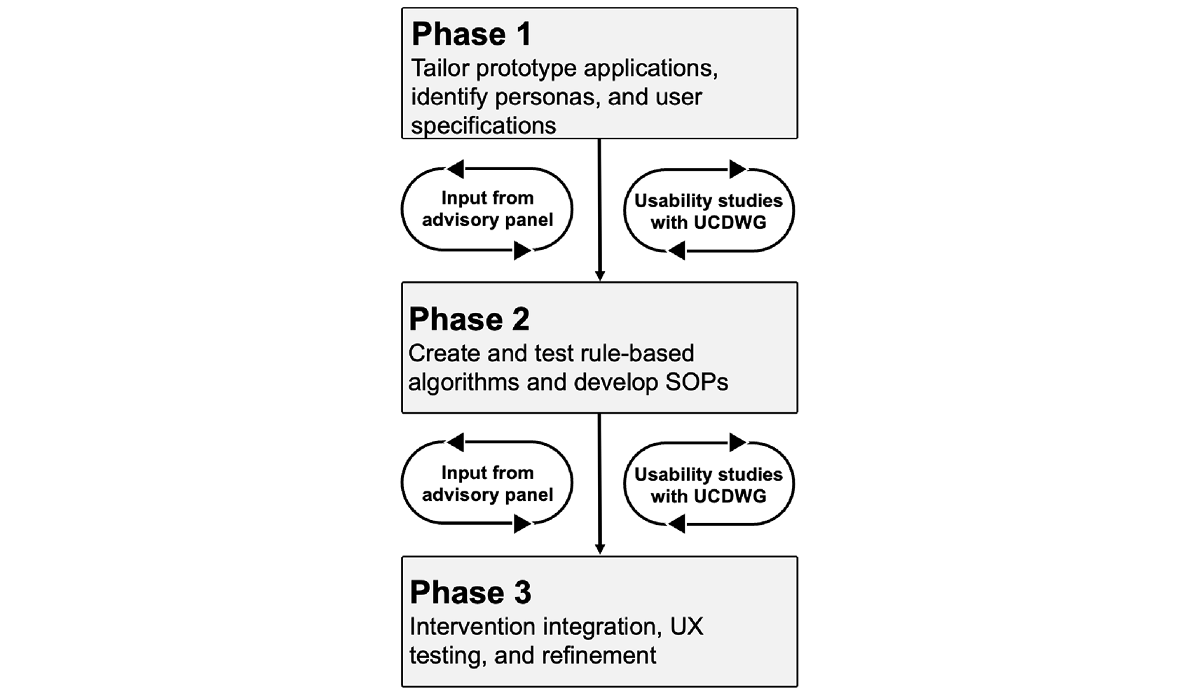
Depiction of the iterative phases of user-centered design activities involved in the ACHIEVE solution. SOP: standard operating procedures; UCDWG: user-centered design work group; UX: user experience.

ACHIEVE UCD activities involve iterative phases that gather feedback from the UCDWG and an advisory group (Figure 2). UCDWG members review multiple versions of the prototype during focus groups and interviews to ensure that aspects of the solution align with personas, which represent examples of individuals who match the study population. The members provide feedback on the features of the app and dashboard, focusing on usability, usefulness for the population, benefits, and barriers to each feature. The key user-centered principles that we emphasize during our activities involve empathy for the user, consistency in design and goals, context of the user, and reducing the cognitive load of the tools. The feedback gathered is used to further refine the app and dashboard to meet the needs of the end users. These activities helped us identify different aspects of personalization: functional (collectively by patient personas and providers), interface (collectively by patient and collectively for types of providers), content (individually and collectively for patient and collectively for types of providers), and distinctiveness (individually and collectively for patient and collectively for types of providers). For example, regarding content-based personalization, patients will receive different education information based on their reported PROs and clinical and social needs history.

**Figure 3:**
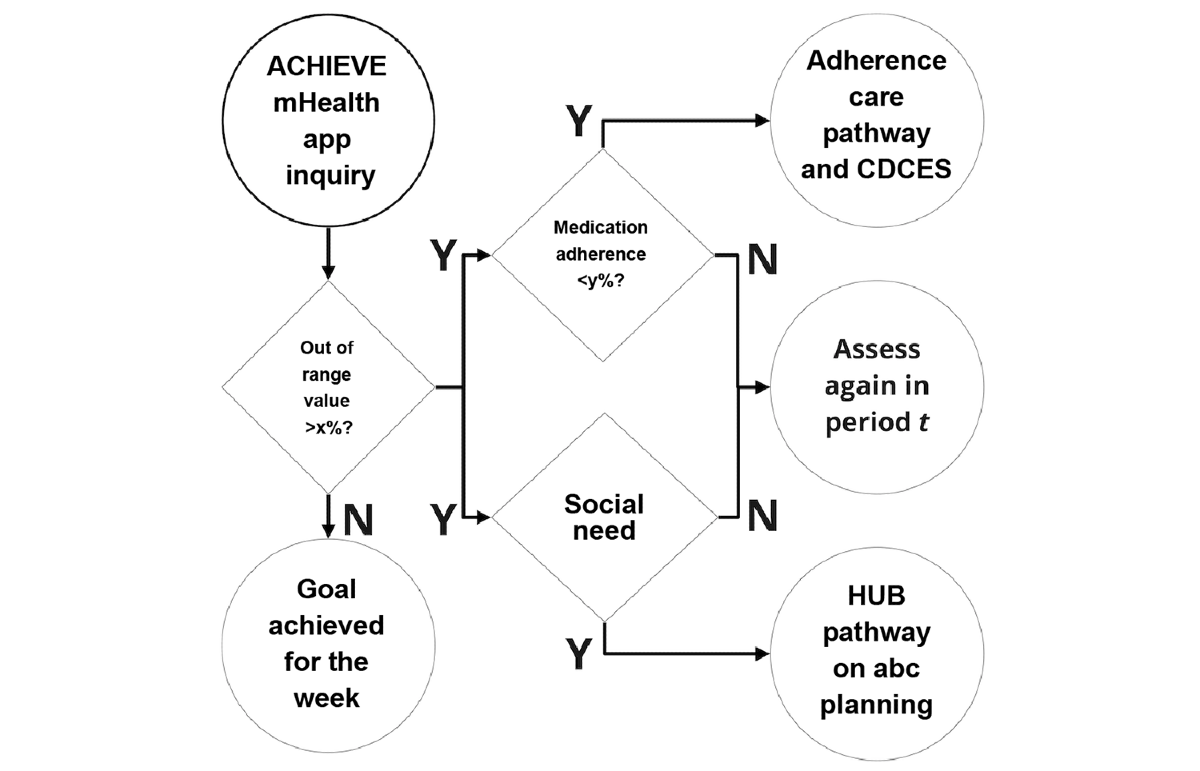
An example rule-based algorithm. CDCES: certified diabetes care and education; HUB: Central Ohio Pathways HUB at Health Impact Ohio; mHealth: mobile health.

User specifications identified during the UCD sessions are used to create and test rule-based algorithms. Inputs for our rules include identification of frequent out-of-range blood glucose values, commonly reported unmet social needs by patients, and care coordination challenges. The purpose of the algorithms is to determine the ideal clinical care (eg, adhere to specific diabetes medication treatment) or social need pathways and PROs to deploy for a specific individual and at a specific time. If a patient has financial constraints to purchase medication, which affects adherence, then a CHW will enroll them in a social needs pathway to address these financial concerns and the algorithm will subsequently assess whether medication adherence and related unmet social needs continue to persist (see Figure 3 for a general example). The algorithms’ recommendations will be evaluated for consistency by CDCES who are a part of the team of providers, using proficiency and efficiency scores [71]. Clinical informatics experts on the study team will conduct a heuristics evaluation of the algorithm based on Bertini’s heuristics tool [72].

Other UCD-based activities include usability testing of the integrated ACHIEVE solution and the use of a modified think-aloud approach that focuses on understanding sociotechnical aspects [73]. The think-aloud approach involves asking members of the UCDWG to respond to task scenarios and open-ended questions while working on the solution. The think-aloud sessions facilitate the identification of informational elements necessary to support effective implementation of technology systems [74,75] and those that preclude user-friendly design [76]. Findings from the think-aloud sessions informed the subsequent refinements to the ACHIEVE solution. Our feasibility study of the ACHIEVE solution prototype, using a UCD approach, indicated a favorable response to the usability of the prototype among both patients who are pregnant and living with diabetes and providers, including maternal fetal-medicine physicians and CDCES [12].

### Design Solution Components Grounded in a Conceptual Framework

**Figure 4:**
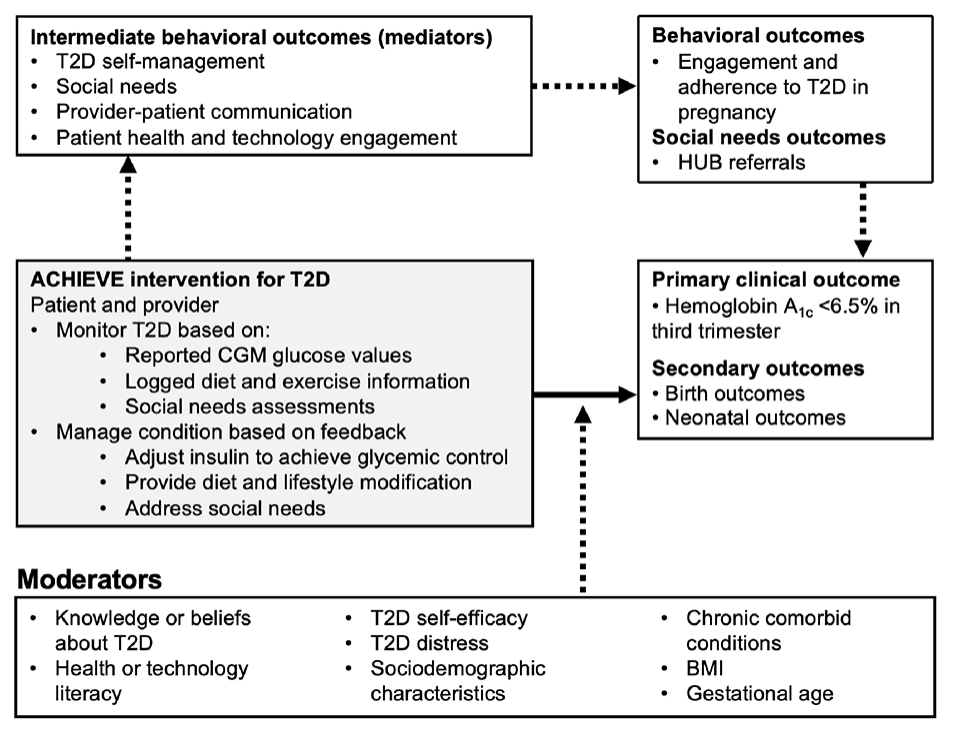
Illustration of the conceptual framework of the ACHIEVE solution for T2D, guided by the Social Cognitive Theory. The model outlines the pathway from monitoring and managing T2D to improved clinical outcomes, influenced by intermediate behavioral outcomes and various moderating factors. CGM: continuous glucose monitoring; HUB: Central Ohio Pathways HUB at Health Impact Ohio; T2D: type 2 diabetes.

We based our solution on a conceptual framework (Figure 4) guided by Social Cognitive Theory [13], which posits that the successful performance of a behavior depends on an individual’s behavioral capability and cognitive and environmental influences on behavior via three domains: (1) skills, (2) knowledge and beliefs, and (3) self-efficacy. Our solution provides patients with educational information that clearly explains how behavior change can achieve a target HbA_1c_ of <6.5% prior to delivery (knowledge and beliefs), which is the primary clinical outcome. The solution develops patients' skills to engage with the diabetes care team by collecting and synthesizing detailed glycemic information via CGM and the provider dashboard to better communicate with the diabetes care team (skills). Team-based coaching that uses CGM data, PROs, and clinical care and social need pathways will help the patient better adhere to T2D care. Closing the loop (eg, having the CHW document the patient securing healthy food through a food pantry) will ensure that a patient’s new skills yield meaningful outcomes and enhance their confidence (self-efficacy). In the context of our study, we work with the Central Ohio Pathways HUB at Health Impact Ohio (HUB) which is a generalizable, regional coordination entity that contracts with care coordination agencies that employ CHWs, who are trained and certified by the HUB, to assess the social needs of individuals and connect them to community resources.

### Design a Multicomponent mHealth Solution

#### Overview

**Figure 5:**
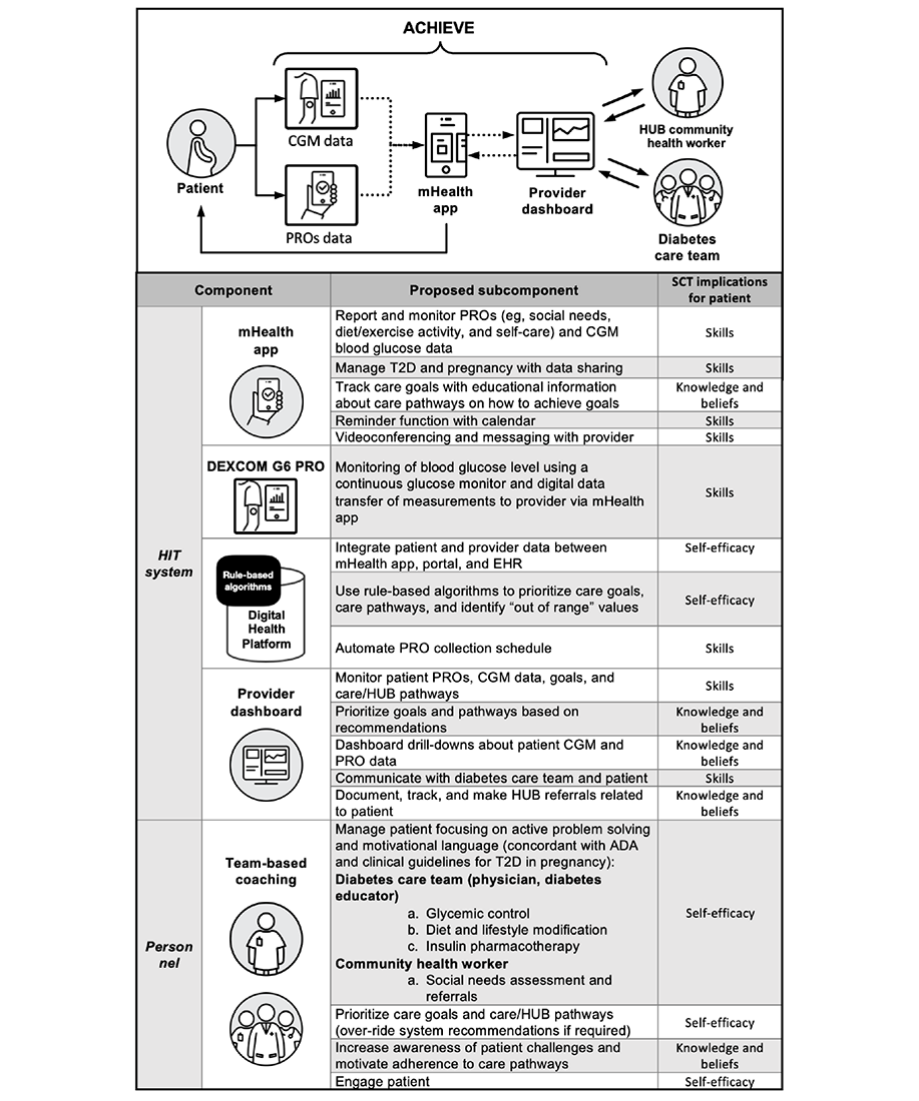
Diagram and table detailing the integrated ACHIEVE solution for managing uncontrolled T2D in Medicaid-eligible pregnant individuals. The system-based solution combines real-time patient-reported outcomes and continuous glucose monitoring data collection via a mHealth app with a provider-facing digital health platform. The system's functionalities, tailored care goals, and pathway recommendations are grounded in clinical guidelines. The table breaks down the components of the system, their subcomponents, and the implications for patients according to the Social Cognitive Theory. ADA: American Diabetes Association; CGM: continuous glucose monitoring; EHR: Electronic Health Record; HIT: Health Information Technology; HUB: Central Ohio Pathways HUB at Health Impact Ohio; mHealth: mobile health; PRO: patient reported outcome; SCT: Social cognitive theory; T2D: type 2 diabetes.

ACHIEVE integrates new and existing technologies to develop an innovative ecosystem for Medicaid-eligible pregnant individuals with uncontrolled T2D. This solution collects real-time patient PRO and CGM data using a mHealth app. Data are transferred to a digital health platform, which displays data on a provider dashboard. Rule-based algorithms facilitate prompt recommendations on care goals and pathways for the patient and provider (Figure 5).

#### mHealth App

The mHealth app provides diverse functions, including education, reminders, clinical care goals, clinical care or social need pathway recommendations, CGM data and PROs reporting and monitoring, messaging and videoconferencing, and a calendar function. Content is based on clinical guidelines for T2D in pregnancy as per the American Diabetes Association and the American College of Obstetricians and Gynecologists [22,77]. Patients will be directed to appropriate resources and web-based learning to help them navigate the app and its resources. PROs in the mHealth app will be embedded to address health and social needs, and rule-based algorithms will provide tailored care goals, show care pathways, and establish the frequency of elicited PROs. PROs, including social needs screening, will be performed through the app (Section 508 compliant). Follow-up questions will use a rule-based algorithm and a pre-established frequency.

#### Continuous Glucose Monitoring Device

Patients will be provided with DEXCOM G6 PRO CGM sensors and transmitters. The Dexcom G6 CGM system is accurate and safe in pregnant individuals with diabetes [78]. Patients will be taught how to place and remove CGM sensors by a nurse and will be given sensors to change themselves at home every 10 days. Of note, the DEXCOM G6 PRO can be applied as a patch on the abdomen, arm, or upper buttocks, is well-tolerated in pregnancy, and does not require calibration [78]. Our mHealth app will allow for wireless synchronization with the CGM sensor so that data are seamlessly reported back to the health care team [79,80].

#### Digital Platform

A robust digital platform will be developed that integrates with Research Electronic Data Capture (REDCap) and algorithms that can adjust the PRO collection protocol based on defined parameters. For instance, if a social need is identified, the platform will automatically increase the frequency of outreach until it is addressed or there is a manual override by a care team member or the patient. This platform allows for the integration of our ACHIEVE mHealth app and dashboard (using a SMART-Fast Health care Interoperability Resources [FHIR] interface). The platform will integrate electronic health record (EHR) data into the provider dashboard and update it over time. It can use rule-based algorithms to synthesize data reported by pregnant individuals and their care team, tailoring care in real time based on changing clinical and social needs. The CHW will integrate their coordination system with the digital platform via a 2-way application programming interface (API) that will allow for the bidirectional communication of structured and unstructured data about a patient’s health status (eg, trends in HbA_1c_ or CGM data such as time in range and comorbid conditions), social needs, and demographics.

#### Provider Dashboard

The ACHIEVE solution will include a bidirectional dashboard that displays information about individuals, including priority care goals and pathways, and recommendations generated via the digital platform. Health care team members can access the dashboard embedded within a portal to modify or update information and close the loop on patient tasks. The dashboard will present recommendations for patient goals and pathways provided by the digital platform algorithms. Providers can use these recommendations or manually select ones for the patient. Providers can sequence goals and pathways by level of complexity. Both the CHW and the health care team can perform ongoing assessments of social need pathway selections and assess recurring needs through the provider dashboard. Documentation of unstructured data will involve information about social need pathways that have not been completed and the reason why, as well as any resources that were used to help support the patients' needs. Unique dashboard views and access to functions will be developed based on the care team role.

#### Community Health Worker and Social Need Pathways

Patients will be screened at enrollment and throughout the solution for social needs using a survey adapted from validated instruments, such as the Accountable Health Communities Health-Related Social Needs Screening Tool. The questionnaire includes 26 questions addressing living situations, food security, transportation, uses, safety, financial strain, employment, family and community support, education, physical activity, substance abuse, mental health, and disabilities [50]. The care team will refer patients with affirmative responses to the HUB through the provider dashboard to address unmet social needs (eg, food insecurity, housing, and employment). HUB CHWs will perform comprehensive social needs assessments and connect patients to community resources through social need pathways, a defined action plan addressing patient needs which is recorded and tracked. For example, this approach will be used to connect a patient who identifies as living in a food desert to a food pantry, which will provide multiple food options and access to fresh produce (trackable by both the CHW and diabetes care team). Linking the digital platform to the CHW is a novel digital health approach using mHealth that can be replicated across health systems. The CHWs can interact with the direct lived experiences of patients as individuals embedded within the community.

#### Team-Based Coaching

Our solution design will involve prespecified roles for health care team members addressing clinical care (physician, CDCES, and nurse) and social needs (CHW) for T2D care in pregnancy. Patients will receive core training by the CDCES to carry out tasks with the tools with the aid of web-based resources and videos. The primary goals of the care team and CHW will be to support the patient with active problem-solving, to motivate engagement with the ACHIEVE solution, and to accomplish care pathway objectives related to glycemic control. The CDCES will be available to patients via videoconferencing and messaging and can message the CHW. Both the CHW and CDCES will monitor the dashboard for social needs. If there is a need identified, a referral will be made to initiate a social need pathway and the patient’s progress will be documented.

#### System Integration

**Figure 6:**
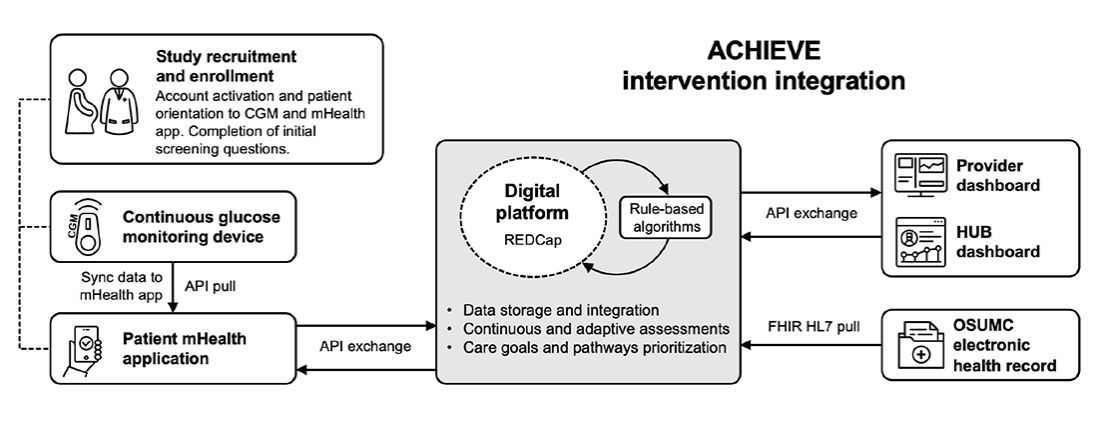
Schematic representation of the data flow within the ACHIEVE solution. The diagram highlights the integration of the CGM device, mHealth app, and EHR system with the digital platform (REDCap) and provider dashboards. API: application programming interface; CGM: continuous glucose monitoring; FHIR: SMART-Fast Health care Interoperability Resources; HL7: Health Language Seven; HUB: Central Ohio Pathways HUB at Health Impact Ohio; mHealth: mobile health; OSUMC: Ohio State University Medical Center; REDCap: Research Electronic Data Capture.

The mHealth app will be updated for the iOS (Apple) and Android (Google; CGM and PROs) operating systems and the EHR will be integrated with the digital platform and the provider dashboard. Health Language Seven and Clinical Quality Language will be used to facilitate integration with the EHR system. Data collection will commence once a patient has concurrently activated their mHealth app and CGM device, as demonstrated in Figure 6. The data are sent in JavaScript Object Notation format to the digital platform using a Representational State Transfer API. The platform has an integrated REDCap web server. The transferred data will be converted to a pre-established schema and stored in a MySQL (Oracle Corporation) database. The required data fields from the EHR will be integrated within the platform using the FHIR protocol for EHR data integration or exchange. Continuous data processing and execution of adaptable rule-based algorithms will be performed using standard R software (R Core Team) packages. The data exchange between the digital platform, mHealth app, and dashboard will occur with the aid of Representational State Transfer APIs at multiple time resolutions (real time, hourly, daily, and weekly). All endpoints and APIs will be secured via transport layer security, and access tokens under the APIs will be used to ensure the secure exchange of data. All users (patients and providers) will access the system for the ACHIEVE solution using an OAuth (version 2.0; Internet Engineering Task Force) compliant identity and access management system.

#### Solution Evaluation Using a Hybrid Implementation Study Design

ACHIEVE involves a hybrid trial type 1 study [81], and consists of 3 aims: aim 1, use UCD practices to adapt our mHealth app solution and provider dashboard to our target population; aim 2, conduct a randomized controlled trial of the solution versus the current standard of care. This trial will assess whether the multicomponent solution (mHealth app with CGM, provider dashboard, and care team coaching) can achieve glycemic control (HbA_1c_<6.5%) by the end of the pregnancy prior to delivery versus current standard care; and aim 3, conduct a multistakeholder evaluation using mixed methods to identify barriers and facilitators to the implementation and uptake of the solution and its subcomponents, including patient and care team engagement, experience, and satisfaction. An effectiveness-implementation hybrid study design, such as ACHIEVE, supports the identification of problems with the delivery of the solution during the clinical trial, which can translate to vital considerations (eg, barriers or facilitators and modifications to maximize uptake and use) for subsequent real-world implementation.

### Limitations to Consider

Technological, end user acceptance, and scalability are examples of challenges that will need to be mitigated through the design, implementation, and evaluation of digital health tools. The use of user-centered design principles and a focus on the context of implementation are 2 examples of approaches that help to systematically address such challenges and these approaches need to be instilled from the beginning of the design process and maintained throughout the study, as suggested by our framework. Some challenges, however, may need to be addressed over the long-term at the policy level (eg, making the internet accessible to everyone in a community, including individuals who face financial barriers). Our framework and the evidence that can be generated from it provide information to raise awareness of these priority issues both at the community and policy levels. Lessons learned from our experience with integration will also form the foundation for additional work that will need to be done to integrate our solution across EHR systems among hospital systems and community-wide.

### Conclusions

We present an evidenced-based framework for mHealth design that is inclusive and personalized for individuals who live with high-risk clinical conditions and experience a high burden of social needs. Our framework is informed by our experiences with designing the multicomponent ACHIEVE solution, including mHealth, a provider dashboard, and team-based coaching, for Medicaid-eligible pregnant individuals with uncontrolled T2D, many of whom experience a high burden of social needs. ACHIEVE addresses prior challenges in using mHealth solutions that include a lack of comprehensive and adaptive evidence-based educational content, closed-loop integration with external sensors, and personalization for individuals who experience obstacles to using a mHealth solution. Our solution moves beyond simple tailoring of mHealth apps based on design specifications typically collected from homogeneous patient populations, including those who do not experience a high burden of adverse SDoH (both clinical and nonclinical) [9]. The ACHIEVE solution will be capable of personalizing care based on shifting clinical and social need contexts for an individual and provide them with dynamic goals to influence their engagement. Our approach presents an opportunity for apps among other populations that experience a high burden of adverse SDoH.

## Abbreviations

API: application programming interface
HbA1c: hemoglobin A1c
CDCES: certified diabetes care and education specialists
CGM: continuous glucose monitoring
CHW: community health worker
EHR: electronic health record
FHIR: SMART-Fast Health care Interoperability Resources
HUB: Central Ohio Pathways HUB at Health Impact Ohio
mHealth: mobile health
PRO: patient-reported outcome
REDCap: Research Electronic Data Capture
SDoH: social determinants of health
SMBG: self-monitoring of blood glucose
T2D: type 2 diabetes
UCD: user-centered design
UCDWG: user-centered design work group
